# Development and Validation of an Isocratic HPLC Method for the Simultaneous Determination of Avobenzone and Tris-Biphenyl Triazine

**DOI:** 10.1021/acsomega.5c12064

**Published:** 2026-03-12

**Authors:** Júlio A. Miranda, Yasmin F. da Cruz, Éverton N. Alencar, Wógenes N. Oliveira, Daniel Cristian F. Soares, Maureen D. Donovan, E. Sócrates T. Egito

**Affiliations:** † Graduate Program in Health Sciences, 28123 Federal University of Rio Grande do Norte, UFRN, Natal 59012-570, RN, Brazil; ‡ Department of Pharmaceutical Sciences and Experimental Therapeutics, College of Pharmacy, University of Iowa, Iowa City, Iowa 52242, United States; § Laboratory of Micro and Nanostructured Systems, College of Pharmaceutical Sciences, Food and Nutrition, 54534 Federal University of Mato Grosso do Sul (UFMS), Campo Grande 79070-900, MS, Brazil; ∥ Laboratory of Bioengineering, Pure and Applied Institute, 28094 Federal University of Itajuba, Itabira 35903-087, MG, Brazil

## Abstract

Avonenzone (AVO) and tris-biphenyl triazine (TBPT) are ultraviolet (UV) filters commonly used in sunscreen formulations. Their combination enables broad-spectrum photoprotection and improved photostability. Despite their widespread and emerging use, the **simultaneous quantitative analysis of these highly hydrophobic compounds remains analytically challenging**, particularly in complex formulation matrices. In this study, an isocratic HPLC method was developed and validated for the simultaneous determination of AVO and TBPT. Chromatographic separation was achieved using a reversed-phase RP-18 column (125 Å, 3.9 mm × 300 mm, 10 μm) with a mobile phase composed of acetonitrile, isopropyl alcohol, and 2% aqueous phosphoric acid (42:42:16, v/v/v). The flow rate was set to 1.0 mL/min, with an injection volume of 20 μL and a column temperature of 30 °C. Detection was performed at 358 nm for AVO and 310 nm for TBPT. Method validation was conducted in accordance with **ICH Q2­(R1)**, including evaluation of specificity, linearity, limits of detection and quantification, accuracy, precision, and robustness. **Method robustness was systematically assessed using a Box–Behnken Design (BBD)** as a Quality-by-Design tool to identify critical chromatographic parameters and define a reliable analytical domain. The method demonstrated excellent linearity over the 0.5–32 μg mL^–1^ range, high accuracy, acceptable precision, and adequate sensitivity for both analytes. Specificity was confirmed using a **representative laboratory-prepared sunscreen formulation**, demonstrating the method’s ability to handle complex matrices without interference from formulation excipients. The BBD analysis confirmed the robustness and reproducibility of the method under small deliberate variations of critical parameters. Overall, the proposed method provides a **simple, reliable, and reproducible isocratic analytical approach** for the simultaneous quantification of AVO and TBPT. Its applicability to complex sunscreen matrices and its emphasis on robustness rather than ultrafast separation make it particularly suitable for **routine quality control, formulation development, and stability studies**, supporting current and future sunscreen research in an evolving regulatory landscape.

## Graphical abstract

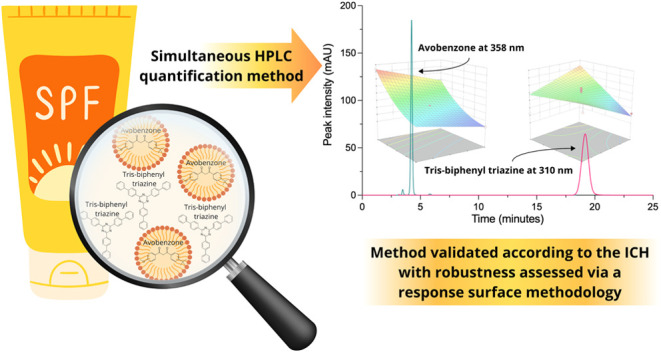

## Introduction

Butyl Methoxydibenzoylmethane, commonly known as avobenzone (AVO), is an organic ultraviolet (UV) filter widely employed in sunscreen formulations. It is highly valued for its ability to absorb radiation in the UVA spectrum (320–400 nm), thereby protecting the skin from long-wave UV exposure associated with photoaging and increased risk of skin cancer.^1^ Despite its remarkable efficacy, particularly within the UVA-I range (340–400 nm), AVO is inherently photounstable, undergoing degradation upon UV exposure that leads to reduced effectiveness and formation of potentially harmful byproducts.^1,2^ Consequently, several strategies have been explored to enhance its photostability, including the incorporation of stabilizing agents and complementary UV absorbers.^3^

A promising stabilizing agent is tris-biphenyl triazine (TBPT), commercially known as Tinosorb A2B, a broad-spectrum UV filter that absorbs across both the UVA-II (320–340 nm) and UVB ranges (290–320 nm).^4^ TBPT enhances AVO’s photostability by providing supplementary absorption and scattering of UVB radiation, thereby acting as a photostabilizer and minimizing AVO degradation.^5^ The combined use of AVO and TBPT in sunscreen formulations therefore offers improved broad-spectrum UV protection and enhanced formulation stability. However, the reliable quantification of these filters during formulation development remains analytically challenging, underscoring the need for precise and validated analytical methods.^6^

Although high-performance liquid chromatography (HPLC) is widely used for quantifying individual UV filters, simultaneous determination of AVO and TBPT using a simple isocratic method has proven difficult due to their distinct chemical characteristics.^7^ AVO, a dibenzoylmethane derivative containing conjugated carbonyl groups attached to aromatic rings (Figure 1), is relatively lipophilic and soluble in nonpolar solvents, influencing its chromatographic retention. Conversely, TBPT exhibits a complex, 3D structure composed of triazine rings and multiple substituted aromatic groups (Figure 1), resulting in high hydrophobicity and low polarity.

**Figure 1:**
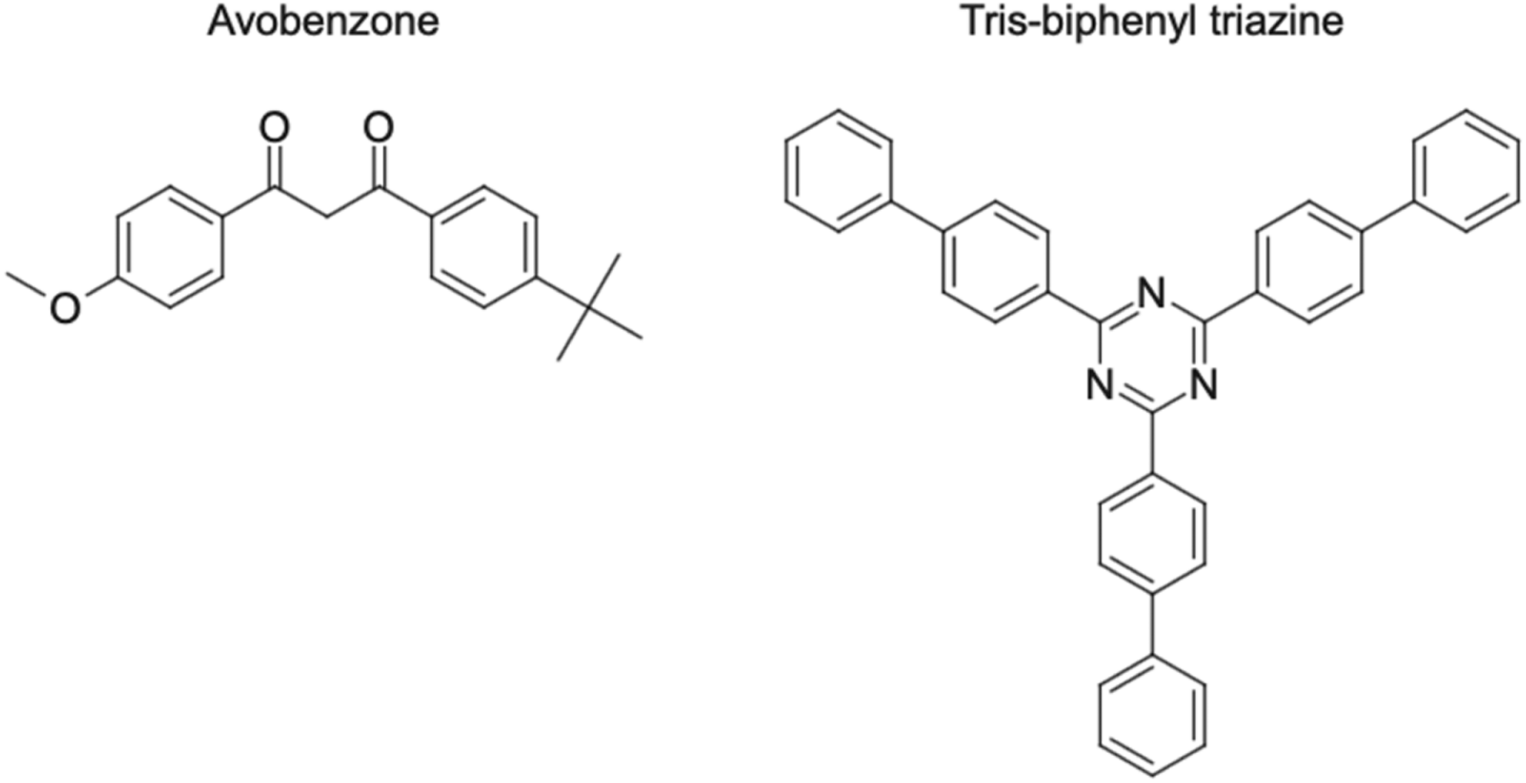
Chemical structures of avobenzone and tris-biphenyl triazine were drawn with MarvinSketch (ChemAxon).

The only simultaneous quantification method currently reported for AVO and TBPT employs gradient elution, and to date, no isocratic HPLC method has been described for their concurrent analysis in pharmaceutical or cosmetic formulations.^8^ Developing such a method would provide a more accessible, cost-effective, and reproducible alternative for routine analysis, enabling:

(i) faster preformulation studies for new AVO and TBPT-containing sunscreens,
(ii) improved analytical tools for quality control, and
(iii) enhanced applicability to *in vitro, ex vivo*, and *in vivo* studies involving photostability, skin permeation, and retention.^8,9^ These aspects are critical for assessing product performance and ensuring regulatory compliance in the development of safe and effective sunscreens.^10^

Given the importance of ensuring method robustness, the application of statistical design tools such as the Box–Behnken Design (BBD) has become increasingly valuable in analytical method development. Integrating BBD into the validation process enables a systematic evaluation of critical analytical parameters, ensuring that the developed method is reliable, accurate, and statistically optimized for diverse formulation studies.^11–13^

To the best of our knowledge, no isocratic HPLC method has been reported for the simultaneous determination of AVO and TBPT. Currently available methods predominantly focus on single UV filters or rely on gradient elution for multicomponent analysis.^8,14,15^ In light of the growing demand for photostable, broad-spectrum sunscreen formulations and the dynamically evolving regulatory landscape for UV filters, there is a clear need for robust and straightforward analytical tools capable of simultaneously quantifying both established and emerging filters, including triazine derivatives.

Therefore, this study aimed to **develop and validate an isocratic HPLC method** for the simultaneous determination of AVO and TBPT. A **Box–Behnken Design** was employed to systematically assess method robustness and to define an analytical design space capable of delivering consistent, reproducible, and reliable results.

## Materials and Methods

### Reagents

Tris-biphenyl triazine (TBPT, Tinosorb A2B) was kindly provided by BASF (São Paulo, SP, Brazil), and avobenzone (AVO) was purchased from Infinity Farma (São Paulo, SP, Brazil). Acetonitrile (ACN) and isopropanol (IPA) (HPLC grade), phosphoric acid (H_3_PO_4_) (P.A.), linseed oil, Span 80, and Kolliphor RH 40 were obtained from Sigma-Aldrich (St. Louis, MO, USA). *N,N*-Dimethylformamide (DMF) (HPLC grade) was obtained from Fisher Scientific (Waltham, MA, USA). A 0.45 mm poly­(tetrafluoroethylene) (PTFE) syringe filter was purchased from MDI Membrane Technologies (Harrisburg, PA, USA). Vitamin E and potassium sorbate were supplied by Engenharia das Essências Comercial LTDA (São Paulo, SP, Brazil), and xanthan gum was obtained from Via Farma (São Paulo, SP, Brazil).

### Instruments

A Hewlett–Packard 8453 UV–visible spectrophotometer (Agilent Technologies, Santa Clara, CA, USA) was used for exploratory analyses. Chromatographic analyses were performed on an Agilent HPLC 1100 series instrument equipped with a photodiode array (PDA) detector and ChemStation software (Version B.04.03) (Agilent Technologies, Wilmington, Germany). Separation was achieved using a Waters μBondapak RP-18 column (125 Å, 3.9 mm × 300 mm, 10 μm particle size).

### Stock Solutions

Primary stock solutions (SS) were prepared at a concentration of 800 μg mL^–1^ for each compound. AVO and TBPT were used as received, with purity certified by the manufacturers, and were considered secondary pharmaceutical standards for the method validation. Each SS was obtained by dissolving 20 mg of AVO or TBPT in a 25 mL volumetric flask filled with DMF. Secondary solutions were prepared by transferring 400 μL of each SS into 10 mL volumetric flasks containing ACN:IPA (1:1, v/v) mixture, resulting in a final concentration of 32 μg/mL for each analyte. All solutions were filtered through a 0.45 μm PTFE syringe filter prior to analysis.

**Exploratory UV–vis** scans were conducted to determine the maximum absorption wavelengths (*l*_max_) for AVO and TBPT. These spectra guided the selection of optimal detection wavelengths for subsequent HPLC analyses.

### Chromatographic Conditions

The optimized isocratic HPLC method employed a mobile phase of acetonitrile, isopropanol, and 2% aqueous H_3_PO_4_ solution (ACN:IPA:2% aqueous H_3_PO_4_ solution) (42:42:16, v/v/v). The flow rate was 1.0 mL min^–1^, with an injection volume of 20 μL and a column temperature maintained at 30 °C. Detection was performed at 310 nm for TBPT and 358 nm for AVO, which were also used for quantification. Each chromatographic run lasted 30 min. All reagents were of HPLC grade.

### System Suitability

System suitability was assessed by six replicate injections of the AVO and TBPT solutions (32 μg mL^–1^). Parameters evaluated included retention time, peak area, peak height, number of theoretical plates, and peak symmetry, ensuring the chromatographic system’s precision and reproducibility.^16^ Suitability was confirmed prior to method validation by following standard acceptance criteria.

### Validation

The analytical method was validated according to the International Conference on Harmonization of Technical Requirements (ICH) for Registration of Pharmaceuticals for Human Use, Q2 (R1) guidelines.^17^ Validation parameters included specificity, linearity, limits of detection (LOD) and quantification (LOQ), precision, accuracy, and robustness^17^ ensuring that the method provided consistent, accurate, and reproducible results for simultaneous analysis of AVO and TBPT.

### Specificity

Specificity was assessed using the medium-level formulation described in the accuracy test as a representative matrix. The evaluation determined whether excipients or formulation components interfered with the detection of AVO or TBPT. Chromatograms were examined to confirm that both analytes produced distinct, well-resolved peaks without overlap or interference.

### Linearity, LOD, and LOQ

The linearity of each analyte was evaluated by the linear regression of three independent calibration curves at seven concentration levels: 0.5, 1.0, 2.0, 4.0, 8.0, 16.0, and 32.0 mg mL^–1^. Each level was prepared by serial dilution of the stock solutions in ACN:IPA (1:1, v/v). All chromatographic peaks were automatically integrated, and the linearity was assessed through correlation (*R*) and determination (*R* ^2^) coefficients.

The limits of detection (LOD) and limits of quantification (LOQ) were calculated according to ICH Q2­(R1) guidelines using the following equations:

1 $$\mathrm{LOD}=\frac{3.3\times s}{S}$$

2 $$\mathrm{LOQ}=\frac{10\times s}{S}$$

where *s* is the standard deviation of the response and *S* is the slope of the calibration curve.

The s values were derived from the regression analyses of three calibration curves using the standard deviation of predicted *y*-values (peak area) across the range of *x*-values (concentrations).

### Precision

Method precision was assessed in terms of intraday and interday variability. Intraday precision was determined by analyzing three independently prepared samples at three different concentration levels (0.5, 4.0, and 32.0 μg mL^–1^). Interday precision was evaluated by repeating the same procedure on three consecutive days. Variations in peak area and retention time were expressed as relative standard deviations (RSD %), which were used to assess method repeatability and reproducibility.

### Accuracy

The accuracy was determined from recovery experiments of three different samples performed at three concentration levels: low (2.60 μg mL^–1^ AVO, and 20 μg mL^–1^ TBPT), medium (3.25 μg mL^–1^ AVO, and 25 μg mL^–1^ TBPT), and high (3.90 μg mL^–1^ AVO, and 30 μg mL^–1^ TBPT). Samples were prepared in a representative UV-Matrix formulation containing linseed oil (1.00%), Kolliphor RH 40 (0.87%), Span 80 (1.13%), vitamin E (1.00%), potassium sorbate (0.20%), AVO (0.80–1.20%), TBPT (6.40–9.60%), and purified water qsp 100%. Recoveries were calculated according to eq 3:

3 $$\mathrm{Recovery}\;\left(\%\right)=\frac{\mathrm{Measured concentration}}{\mathrm{Theoretical concentration}}\times 100$$

### Production of UV-Matrix

The laboratory-prepared nanoemulsified UV-Matrix was intentionally designed to realistically replicate the compositional complexity of commercial sunscreen formulations and evaluate potential matrix interferences during chromatographic analysis. The nanoemulsion was prepared by the phase inversion composition method. The oil phase, comprising linseed oil, AVO, vitamin E, Kolliphor RH 40, and Span 80, was mixed under magnetic stirring at 50 °C. The aqueous phase, consisting of purified water, TBPT, and potassium sorbate, was prepared under identical conditions. After 10 min, the aqueous phase was added dropwise to the oil phase under constant stirring, and the resulting mixture was stirred for an additional 15 min until complete emulsification was achieved.

### Robustness Using a Box–Behnken Design

A Box–Behnken Design with three factors at three levels each was employed as a robustness-centered QbD tool to systematically investigate critical chromatographic parameters, including organic solvent content, mobile phase flow rate, and column temperature. This experimental design was selected for its ability to efficiently estimate main effects and interaction terms with a relatively small number of experimental runs (*n* = 17), while maintaining adequate statistical power.^12,18,19^

The independent variables are as follows: (A) proportion of organic solvent (ACN:IPA 1:1) in the mobile phase (82–86%), (B) column oven temperature (25–35 °C), and (C) mobile phase flow rate (0.9–1.1 mL min^–1^). The dependent variables (responses) were: (R1) retention time, (R2) number of theoretical plates, (R3) peak area, and (R4) peak symmetry. A total of 17 experimental runs were conducted, and the effects of the independent variables on each response were analyzed using Design-Expert software (version 13.0, Stat-Ease). Statistical significance was assessed by analysis of variance (ANOVA) at *p* < 0.05. Coefficients of determination (*R* ^2^) and *F*-tests were used to evaluate the model adequacy and predictive performance. Quadratic model outputs and surface-response plots were generated to visualize parameter interactions and to define robustness domains within the analytical design space.

## Results and Discussion

To date, no analytical methodology has been reported in the literature for the simultaneous isocratic determination of AVO and TBPT. Existing HPLC methods typically address each compound individually, employing aqueous–organic mobile phases or relying on gradient elution techniques to achieve satisfactory separation.^8^ Although gradient methods are effective, they involve more complex solvent systems, increased runtimes, and higher operational costs when compared with isocratic approaches.^20^ Therefore, this study aimed to fill this methodological gap by developing a simple, cost-effective, and robust isocratic HPLC method capable of simultaneously identifying and quantifying AVO and TBPT from a complex formulation matrix (UV-Matrix) in a single analytical run.

Prior to method validation, UV–vis spectrophotometric scans were performed to determine the maximum absorption wavelength (*l*_max_) values for each molecule. The spectra revealed *l*_max_ values of 358 nm for AVO and 310 nm for TBPT (Figure 2), which are consistent with previously reported data.^4,21^ Although UV–vis spectrophotometry lacks the separation capability necessary for analyzing complex mixtures, it remains an essential preformulation tool, providing valuable information for wavelength selection and optimization of chromatographic detection parameters.

**Figure 2:**
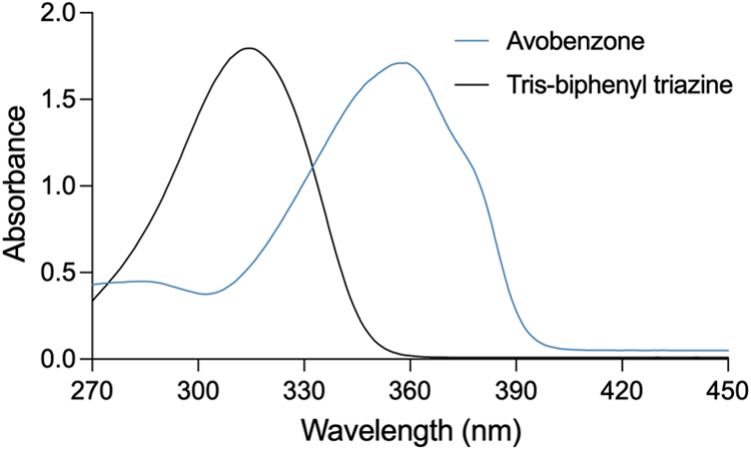
UV–vis spectra of avobenzone and tris-biphenyl triazine recorded in an acetonitrile:isopropanol (1:1) solvent system, showing *l*_max_ values at 358 and 310 nm, respectively.

The recorded spectra of both molecules (Figure 2) exhibited a crossover section at 332 nm. Although this wavelength could, in theory, be used for the simultaneous detection of both compounds, such an approach becomes unreliable when large concentration differences exist between the analytes. In the current study, the concentration of AVO was approximately one-eighth that of TBPT in the target formulation matrix, making separate detection wavelengths more appropriate for accurate quantification.

Following the determination of the *l*_max_ values, exploratory chromatographic trials were conducted to identify a suitable mobile phase composition. Various solvent systems were tested, including methanol:water, acetonitrile:water, isopropanol:water, isopropanol:acetonitrile:water, and isopropanol:methanol:water, in different proportions. However, initial attempts failed to elute TBPT within a reasonable retention time or resulted in asymmetrical peak shapes, with significant tailing or fronting for both analytes.

Adjusting the aqueous phase to pH 3 with 2% (v/v) phosphoric acid (H_3_PO_4_) markedly improved peak symmetry and reproducibility. This improvement can be attributed to the suppression of secondary interactions between the analytes and the residual silanol groups of the stationary phase, minimizing peak distortion caused by partial ionization and improving overall chromatographic performance by stabilizing the analytes’ ionization states at acid pH.^16^

The optimized mobile phase, consisting of ACN:IPA:2% aqueous H_3_PO_4_ (42:42:16, v/v/v), produced sharp, symmetrical peaks with well-resolved retention times of 4.24 min for AVO and 19.18 min for TBPT, as shown in Figure 3.

**Figure 3:**
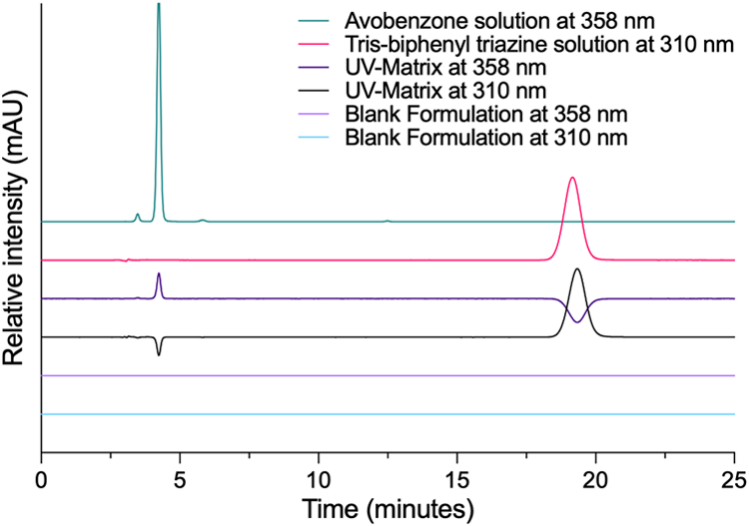
Representative chromatograms of avobenzone (AVO) and tris-biphenyl triazine (TBPT) obtained during the specificity study at detection wavelengths of 310 and 358 nm. The traces correspond to individual AVO and TBPT solutions, the combined formulation containing both analytes (UV-Matrix), and the blank formulation. Minor downward deflections observed in some overlaid traces arise from baseline offset and signal-scaling artifacts introduced by multiwavelength visualization using ChemStation software (version B.04.03) and do not represent analytical peaks or negative absorbance.

An essential preliminary step before full method validation is to confirm that the chromatographic system consistently delivers reproducible results for repeated injections of the same sample. This evaluation ensures that the analytical setup, including the HPLC system, column, and detector, operates under stable and controlled conditions. The system suitability parameters, summarized in Table 1, demonstrate the equipment’s capability to generate precise and reliable chromatographic performance for both analytes. All measured values met the acceptance criteria established by ICH guidelines, confirming the system’s adequacy for subsequent validation steps.

**Table 1:** System Suitability Parameters for Avobenzone and Tris-Biphenyl Triazine Determined from Six Replicate Injections of 32 μg mL^–1^ Standard Solutions^a^

Analyte	Parameters	Average (*n* = 6)	RSD (%)
Avobenzone	Retention time (min)	4.24	0.07
	Peak area (mAU·min)	1761.91	0.80
	Peak height (mAU)	185.73	0.64
	Theoretical plates	13251.70	2.14
	Peak symmetry	0.98	2.79
Tris-biphenyl triazine	Retention time (min)	19.18	0.07
	Peak area (mAU·min)	2801.93	0.72
	Peak height (mAU)	64.36	0.68
	Theoretical plates	13012.89	1.35
	Peak symmetry	1.02	0.57

a RSD: relative standard deviation.

All parameters evaluated in the system suitability test were within the acceptable limits recommended by ICH Q2­(R1), confirming the reliability and precision of the chromatographic system. The relative standard deviation (%RSD) values for retention time, peak area, and peak height were below 1% for both analytes, demonstrating excellent reproducibility of injections and system stability. Theoretical plate counts exceeded 13,000 for both AVO and TBPT, indicating high column efficiency, while symmetry factors (0.98 for AVO and 1.02 for TBPT) were close to unity, reflecting well-shaped, symmetrical peaks without significant tailing or fronting. Collectively, these results confirm that the HPLC system performed consistently and that the selected chromatographic conditions are suitable for the precise quantitative analysis of both UV filters.

All validation parameters were thoroughly assessed in accordance with ICH Q2­(R1), including specificity, linearity, limits of detection and quantification, accuracy, precision (intra- and interday), and robustness. To further support the validation of the experimental design, robustness evaluation was complemented by an extended statistical analysis based on the **Box–Behnken Design (BBD)**. This analysis included analysis of variance (ANOVA), coefficients of determination (*R* ^2^ and adjusted *R* ^2^), lack-of-fit testing, and assessment of adequate precision, confirming the suitability and reliability of the experimental domain explored.

Method specificity was evaluated by comparing chromatograms (Figure 3) obtained from (i) standard solutions of AVO and TBPT, (ii) a formulation containing both UV filters (UV-Matrix), and (iii) a blank formulation without analytes. Under these conditions, only peaks corresponding to the target compounds were detected in UV-Matrix, confirming the absence of interference from formulation excipients at the respective retention times.

As shown in Figure 3, the chromatograms of the UV-Matrix revealed well-resolved peaks for AVO (4.2 min) and TBPT (19.2 min), with no overlapping or unexpected signals within the same retention-time window. The blank formulation chromatogram further confirmed the absence of interfering peaks, supporting the method’s specificity toward the analytes of interest. Minor peaks detected in the AVO chromatogram at 3.48 and 5.82 min were attributed to raw material impurities. Nevertheless, resolution factors above 1.5 ensure complete separation from the target peak, with no analytical interference.^22^

Following the specificity evaluation, the linearity was investigated to establish the quantitative reliability of the method. Calibration curves were constructed over the concentration range of 0.5–32 μg mL^–1^ for both analytes, yielding correlation coefficients (*R*) and determination coefficients (*R* ^2^) ≥ 0.999. The resulting regression equations (Table 2) were subsequently applied to determine the concentrations of AVO and TBPT during the accuracy testing.

**Table 2:** Method Validation Data for Linearity, Limits of Quantification and Detection, and Accuracy for Avobenzone and Tris-Biphenyl Triazine

		Avobenzone	Tris-biphenyl triazine
Linearity	Analytical curve	*y* = 53.56*x* + 7.35	*y* = 93.32*x* + 6.14
	Correlation coefficient (*R*)	0.99996	0.99996
	Linear regression coefficient (*R* ^2^)	0.9999	0.9999
	Limit of detection (μg mL^–1^)	0.49	0.50
	Limit of quantification (μg mL^–1^)	1.49	1.50
Accuracy	Low level	107.99% ± 4.78%	96.81% ± 4.08%
	Medium level	107.94% ± 2.85%	95.76% ± 3.04%
	High level	110.15% ± 1.99%	97.83% ± 1.82%

The calculated limits of detection (LOD) and quantification (LOQ) were 0.49 and 0.50 μg mL^–1^ for AVO and 1.49 and 1.50 μg mL^–1^ for TBPT, respectively. These values were estimated from calibration curves using the standard deviation of the response and the slope in accordance with ICH Q2­(R1), and were further corroborated by the analysis of samples at concentrations near these limits (0.5, 1.0, and 2.0 μg mL^–1^). Representative chromatograms corresponding to these concentrations are provided in the Supporting Information (Figure S1).

The validation results summarized in Table 2 confirm that the developed HPLC method enables reliable and sensitive quantification of avobenzone (AVO) and tris-biphenyl triazine (TBPT). Calibration curves for both analytes were highly linear over the concentration range of 0.5–32 μg mL^–1^, with correlation and determination coefficients (*R* = 0.99996; *R* ^2^ = 0.9999) indicating excellent proportionality between analyte concentration and detector response. The low limits of detection (0.49–0.50 μg mL^–1^) and quantification (1.49–1.50 μg mL^–1^) values demonstrate the method’s suitability for trace-level analysis in formulation studies. Accuracy testing yielded recoveries ranging from 95.7 to 110.1%, all within acceptable limits according to ICH Q2 (R1), confirming adequate trueness and reproducibility. The slightly higher recoveries observed for AVO may be attributed to its greater solubility and extraction efficiency from the oil-based matrix, whereas TBPT recoveries remained consistently close to 100%. Overall, these results demonstrate that the proposed isocratic HPLC method is accurate, precise, and suitable for the simultaneous quantification of AVO and TBPT in complex cosmetic or pharmaceutical matrices.

The accuracy of the method was evaluated by spiking known amounts of AVO and TBPT into the UV-Matrix formulation. Because the assessment was performed using the actual formulation matrix, it also served as a proof of concept for the method’s applicability in complex systems. Recovery values (Table 2) ranged from 95.7 to 110.1%, slightly exceeding the typical ±2% deviation for some concentration levels,^16^ yet remaining within the acceptance criteria recommended by ICH Q2 (R1). The observed variability can be attributed to the inherent complexity of the oil-based nanoemulsified matrix, particularly with respect to the analyte solubilization and extraction efficiency. Nevertheless, the overall recovery profile confirms the **accuracy, robustness, and reliability** of the method for routine quantitative analysis of AVO and TBPT in formulation studies. Following application of the proposed sample-preparation procedure, the resulting AVO and TBPT concentrations consistently fell within the validated linear range of the method, and **no additional dilution was required prior to chromatographic analysis**.

Inter- and intraday precision were evaluated at three concentration levels (0.5, 4 μg/mL, and 32 μg mL^–1^) for both analytes. Although international regulatory agencies such as the European Medicines Agency (EMA), the United States Food and Drug Administration (FDA), and the Brazilian Health Regulatory Agency (ANVISA) do not define fixed acceptance limits for precision, they require that relative standard deviations (RSD %) demonstrate consistency with the method’s intended purpose and overall reproducibility.^23–25^

Although the aforementioned regulatory agencies do not specify a fixed RSD% acceptance criterion, Horwitz (1980) established an empirical relationship demonstrating that analytical variability increases as analyte concentration decreases.^26^ This concept, widely adopted by AOAC International, introduced the use of the Horwitz ratio (HorRat) as a normalized metric across different concentration levels. In validation studies spanning food, pharmaceutical, and agriculture analyses,^27–29^ HorRat values below 2.0 are generally recognized as indicative of consistent and reproducible analytical performance.

In this work, the precision results met those expectations. The observed RSD % values increased at the lowest concentration levels, as predicted by Horwitz’s empirical model, which correlates higher variability with lower analyte concentration. To further verify consistency, the Horwitz ratio (HorRat) was calculated, and all values ranged from 0.12 to 0.91, remaining well below the internationally accepted threshold of 2.0 (Table 3). These findings confirm the adequacy of the developed HPLC method in terms of precision and reproducibility across the evaluated concentration range.

**Table 3:** Validation Data for Intraday and Interday Precision^a^

		Interday			Intraday		
Analyte	Concentration (μg mL^–1^)	Peak area	RSD (%)	HorRat	Peak area	RSD (%)	HorRat
Avobenzone	0.5	30.05	4.50	0.26	29.21	3.90	0.23
	4	228.02	3.45	0.28	224.76	4.53	0.12
	32	1767.01	2.10	0.23	1763.56	2.81	0.31
Tris-biphenyl triazine	0.5	48.86	11.89	0.69	43.43	10.73	0.63
	4	383.86	11.31	0.90	384.03	8.84	0.71
	32	3028.21	8.32	0.91	2995.99	6.23	0.68

a HorRat: Horwitz Ratio; RSD: relative standard deviation.

Based on the results obtained for linearity, LOD, LOQ, precision, and accuracy, the working range for the method was established between 1.50 and 32 μg mL^–1^ for both AVO and TBPT. Although the detection limits were approximately 0.5 μg mL^–1^ for both analytes (0.49 μg mL^–1^ for AVO and 0.50 μg mL^–1^ for TBPT), the quantification limits (1.49 μg mL^–1^ for AVO and 1.50 μg mL^–1^ for TBPT) defined the lower boundary for reliable quantitative analyses. This range fulfills international method validation requirements and ensures the method’s applicability for quality control and formulation development studies in pharmaceutical and cosmetic matrices.

Robustness validation for HPLC methods follows ICH guidelines, which recommend evaluating the influence of minor, deliberate variations in method parameters to verify that analytical performance remains unaffected.^30^ In addition to this conventional approach, a Box–Behnken Design (BBD) was employed to assess robustness in a more systematic and statistically efficient manner, as previously reported by other authors.^12,18,19,30^ The use of BBD enables the simultaneous evaluation of individual and interactive effects among critical variables, reducing the number of experimental runs while providing greater insight into method stability.

In this study, the BBD approach allowed optimization and robustness assessment of three independent factors: (i) the percentage of the organic phase (ACN:IPA, 1:1) in the mobile phase, (ii) the HPLC oven temperature, and (iii) the flow rate. The responses (dependent variables) evaluated included the retention time, theoretical plates, peak area, and peak symmetry.

A sample mixture containing 32 μg mL^–1^ of each analyte (AVO and TBPT) was used across all 17 experimental runs following the BBD matrix. In all conditions, both analytes were successfully separated and quantified without interference, confirming the method’s robustness and specificity under the tested experimental variations. The complete data set obtained from the design is summarized in Table 4.

**Table 4:** Robustness Evaluation Responses for Tris-Biphenyl Triazine and Avobenzone Based on Box–Behnken Design^a^

	Factors			Tris-biphenyl triazine responses				Avobenzone responses			
Execution number	A	B	C	R1	R2	R3	R4	R1	R2	R3	R4
1	84	35	0.9	20.754	14383.362	2291.026	0.996	4.676	13682.708	1490.742	1.017
2	82	30	1.1	23.709	11808.991	1877.191	1.019	4.102	11738.696	1207.060	1.076
3	82	25	1.0	27.642	12912.175	2061.157	0.973	4.553	12136.156	1323.573	1.075
4	86	25	1.0	16.806	13198.638	2141.117	0.997	4.219	15427.142	1356.798	1.016
5	84	25	0.9	22.008	12589.144	2378.156	1.011	4.665	11956.794	1509.808	1.075
6	82	35	1.0	24.957	13657.153	2121.142	1.011	4.450	10477.938	1249.100	1.107
7	86	30	0.9	17.518	14757.146	2403.591	0.991	4.557	14067.989	1514.691	1.027
8	84	30	1.0	18.947	12723.061	2163.073	0.999	4.223	12497.790	1370.196	1.071
9	84	25	1.1	17.905	10915.753	1972.050	1.009	3.853	11913.753	1251.834	1.075
10	82	30	0.9	28.695	13864.410	2387.197	1.027	4.828	10838.438	1530.530	1.061
11	84	35	1.1	16.848	12893.631	1934.899	0.982	3.862	12826.725	1228.277	1.037
12	86	35	1.0	15.321	14326.968	2123.915	0.973	4.080	14485.081	1338.099	1.016
13	86	30	1.1	14.302	12411.739	1937.742	0.982	3.688	12719.977	1221.864	1.034
14	84	30	1.0	19.054	12442.655	2192.642	1.004	4.226	12582.369	1387.241	1.058
15	84	30	1.0	19.214	12548.974	2173.346	1.007	4.233	12497.300	1375.006	1.060
16	84	30	1.0	19.134	12568.292	2177.114	1.006	4.231	12558.262	1378.269	1.058
17	84	30	1.0	19.060	12567.520	2186.866	1.003	4.227	12564.148	1384.080	1.055

a A: Organic solvents (acetonitrile:isopropanol 1:1) concentration (%) in the mobile phase; B: oven temperature (°C); C: mobile phase flow rate (mL min^–1^); R1: retention time in minutes; R2: number of theoretical plates; R3: peak area; R4: peak symmetry.

The results summarized in Table 4 demonstrate that minor variations in the organic phase concentration, column oven temperature, and mobile phase flow rate produced consistent chromatographic responses for both avobenzone and tris-biphenyl triazine. The retention times remained stable, with deviations below 10%, and the theoretical plate counts were consistently above 10,000 for both analytes, confirming high column efficiency throughout all runs. Peak area and symmetry factors also showed minimal fluctuations, with RSD < 2% across comparable conditions, indicating that detector response and peak shape were unaffected by the tested variations. Altogether, these findings support that the analytical method maintains its precision and selectivity within the studied parameter range, confirming its robustness and suitability for routine quality control and formulation analyses.

Multivariate analysis allows evaluating how individual factors and their interactions influence response variables, which in turn helps to define an operating range based on experimental evidence.^31^ In addition, response surface plots built from linear and quadratic models make it possible to visualize these effects and support the optimization method.^30,32^ The data were analyzed using analysis of variance (ANOVA) to verify the model significance, and the statistically significant effects are presented in Table 5 for TBPT and Table 6 for AVO.

**Table 5:** Statistical Data for Tris-Biphenyl Triazine Robustness

Dependent variables	Independent variables	Sum of squares	*F*-Value	*p*-Value	Prob > *F*
R1—Retention time	A—% Organic solvent in the mobile phase	210.70	3086.65	<0.0001	Significant
	B—HPLC oven temperature (°C)	5.25	76.93	<0.0001	Significant
	C—Mobile phase flow rate (mL/min)	32.85	481.23	<0.0001	Significant
R2—Number of theoretical plates	A—% Organic solvent in the mobile phase	7.514 × 10^05^	7.32	0.0304	Significant
	B—HPLC oven temperature (°C)	3.984 × 10^06^	38.81	0.0040	Significant
	C—Mobile phase flow rate (mL/min)	7.152 × 10^06^	69.67	<0.0001	Significant
R3—Peak area	A—% Organic solvent in the mobile phase	3187.12	2.29	0.1738	Not significant
	B—HPLC oven temperature (°C)	830.24	0.5971	0.4650	Not significant
	C—Mobile phase flow rate (mL/min)	3.776 × 10^05^	271.56	<0.0001	Significant
R4—Peak symmetry	A—% Organic solvent in the mobile phase	0.0010	7.64	0.0280	Significant
	B—HPLC oven temperature (°C)	0.0001	0.7521	0.4146	Not significant
	C—Mobile phase flow rate (mL/min)	0.0001	1.06	0.3374	Not significant

**Table 6:** Statistical Data for Avobenzone Robustness

Dependent variables	Independent variables	Sum of squares	*F*-Value	*p*-Value	Prob > *F*
R1—Retention time	A—% Organic solvent in the mobile phase	0.2413	191.35	<0.0001	Significant
	B—HPLC oven temperature (°C)	0.0062	4.89	0.0626	Not Significant
	C—Mobile phase flow rate (mL/min)	1.3000	1028.52	<0.0001	Significant
R2—Number of theoretical plates	A—% Organic solvent in the mobile phase	1.656 × 10^07^	24.90	0.0016	Significant
	B—HPLC oven temperature (°C)	186.31	0.0003	0.9871	Not significant
	C—Mobile phase flow rate (mL/min)	2.267 × 10^05^	0.3410	0.5576	Not significant
R3—Peak area	A—% Organic solvent in the mobile phase	1835.79	3.61	0.0990	Not significant
	B—HPLC oven temperature (°C)	2305.04	4.54	0.0706	Not significant
	C—Mobile phase flow rate (mL/min)	1.615 × 10^05^	318.06	<0.0001	Significant
R4—Peak symmetry	A—% Organic solvent in the mobile phase	0.0064	15.37	0.0057	Significant
	B—HPLC oven temperature (°C)	0.0005	1.25	0.3006	Not significant
	C—Mobile phase flow rate (mL/min)	0.0002	0.4973	0.5035	Not significant

For TBPT, the ANOVA showed that the three independent variables (A, organic phase %; B, oven temperature; and C, flow rate) all affected retention time and theoretical plates. However, the peak area was influenced only by factor C (flow rate), and peak symmetry was influenced only by factor A (organic phase composition).

For AVO, the individual analyses indicated that none of the responses were influenced by all parameters simultaneously. Retention time was mainly affected by factors A and C, whereas theoretical plates and peak symmetry were significantly influenced only by factor A. In turn, factor C also affected the peak area.

The statistical outcomes indicate that the chromatographic behavior of both analytes is primarily influenced by the **organic phase composition (factor A)** and the **flow rate (factor C)**, while the **oven temperature (factor B)** plays a comparatively minor role. The strong dependence of retention time on factors A and C reflects the polarity contrast between the analytes and the mobile phase: slight variations in organic content or flow rate substantially modify elution strength, thereby affecting both analytes’ migration through the stationary phase. The influence of factor A on symmetry parameters suggests that the solvent composition affects analyte–stationary phase interactions, particularly for TBPT, which has a larger, more hydrophobic structure. In turn, factor C’s impact on peak area highlights the sensitivity of detector response to flow variations. Overall, these findings confirm that within the experimental range tested, the method maintains robustness, showing predictable and statistically significant responses to controlled variations without compromising analytical reliability.

Retention time reflects the strength of the interactions between analytes and the stationary phase.^33^ For TBPT, the retention time decreased markedly with increasing flow rates due to the reduced contact time between the analyte and the stationary phase. As a larger and more nonpolar molecule than AVO, TBPT exhibits stronger hydrophobic interactions with the stationary phase. However, at higher flow rates, the diminished interaction time shortens its elution. In contrast, AVO, being smaller and more polar, shows limited sensitivity to changes in the flow rate, eluting more consistently under varied conditions.

Theoretical plates represent chromatographic efficiency, reflecting the number of discrete equilibrations between the stationary and mobile phases.^34^ For TBPT, higher theoretical plate counts were observed at lower flow rates and higher organic phase concentrations. Lower flow rates allow the bulky TBPT molecule to achieve more complete interactions with the stationary phase, enhancing the separation efficiency. Conversely, higher flow rates reduce the equilibration time, causing peak broadening and decreased plate numbers. AVO displayed a less pronounced dependence on these variables; its smaller size and lower hydrophobicity promote rapid, uniform partitioning between the phases. For both analytes, increasing the organic solvent content improved the chromatographic efficiency by minimizing matrix effects and promoting sharper, more symmetrical peaks.

Peak area, a critical parameter for quantitative analysis, was significantly affected by flow rate (*p* < 0.0001). At higher flow rates, the shorter residence time within the stationary phase reduces the number of analyte molecules reaching the detector at any given moment, resulting in smaller peak areas.^35^ TBPT showed greater variability due to its larger molecular structure and stronger stationary phase interactions, whereas AVO’s smaller, more mobile structure produced a more consistent detector response across conditions.

Peak symmetry is another essential parameter influencing the quantification accuracy and resolution. Asymmetric peaks may arise from secondary interactions, such as analyte adsorption, column overloading, or incomplete equilibration.^36,37^ For TBPT, peak symmetry was modestly affected by the organic phase composition but remained within acceptable limits (0.973 to 1.027). This variation can be attributed to secondary interactions at higher organic concentrations, where solvent molecules compete for adsorption sites in the stationary phase. AVO exhibited minimal sensitivity to any of the tested factors; its smaller size and reduced polarity led to fewer secondary interactions. Consequently, its peak shape remained highly symmetric, showing little influence from variations in the flow rate or temperature.

Although the oven temperature had a less statistically significant effect on most responses, its influence on analyte viscosity and diffusivity cannot be disregarded. A temperature of 30 °C provided a suitable balance between efficient mass transfer and stable analyte-stationary phase interactions, particularly for TBPT. Deviations from this condition could lead to subtle changes in elution profiles, especially for larger and more hydrophobic molecules.^38^

Overall, these results demonstrate that the analytical method maintains a stable and predictable performance across deliberate variations in the organic phase composition, flow rate, and oven temperature. Although TBPT exhibited greater sensitivity to parameter changes—particularly in retention time, theoretical plates, and peak area, AVO showed a more consistent and robust chromatographic behavior, reflecting its smaller size and reduced hydrophobicity. Importantly, the variations observed across the evaluated ranges did not compromise analyte identification or quantification, confirming that the method operates reliably under typical laboratory fluctuations. This foundational robustness supports the subsequent evaluation of secondary interactions between factors and their combined influence on chromatographic responses.

Some factors also exhibited significant secondary interactions (*p* < 0.05). For TBPT, two interactions were statistically relevant: A*C for retention time (R1) (*p* = 0.0116) and A*B for peak symmetry (R4) (*p* = 0.0273). These interactions are illustrated in the 3D response surface plots shown in Figure 4.

**Figure 4:**
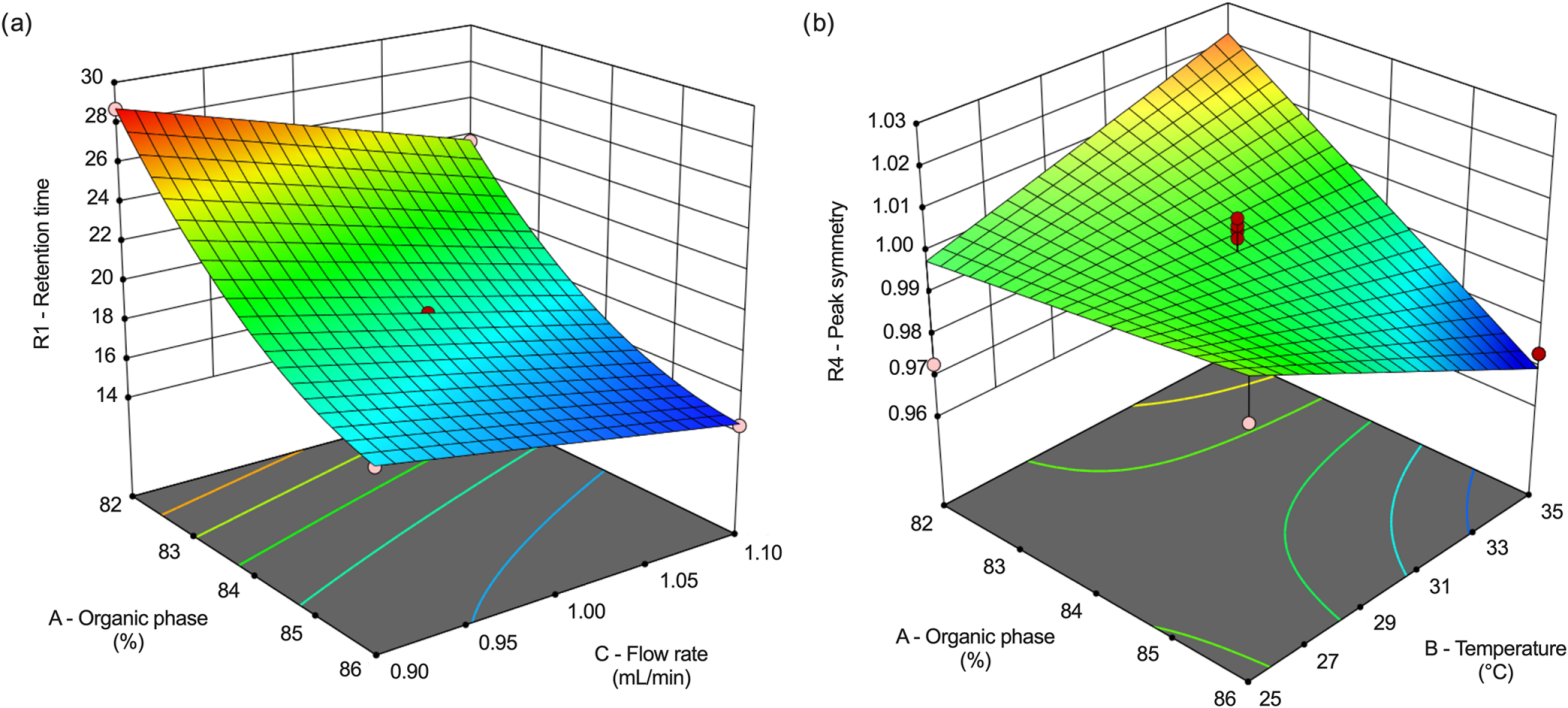
Three-dimensional (3D) response surface plots showing the interaction effects of selected factors on Tris-biphenyl triazine chromatographic responses: (a) interaction between factors A and C on retention time (R1), and (b) interaction between factors A and B on peak symmetry (R4). Factors A: organic solvent concentration (%) in the mobile phase (acetonitrile:isopropanol 1:1); B: oven temperature (°C); C: mobile phase flow rate (mL min^–1^).

In Figure 4a, the interaction between factors A (organic phase percentage) and C (flow rate) on R1 demonstrates that TBT retention time decreases when both the organic content and flow rate increase. This behavior indicates that less polar mobile phase conditions, combined with higher flow rate velocity, enhance elution strength and reduce analyte-stationary phase interaction time, promoting faster elution of TBPT.

Figure 4b depicts the A and B interactions (organic phase percentage × oven temperature) affecting the peak symmetry (R4). Improved symmetry is observed under higher organic solvent percentages and lower oven temperatures, suggesting that stronger elution conditions coupled with milder thermal environments yield more symmetrical peaks. In contrast, low organic solvent levels combined with elevated temperatures reduce peak symmetry, likely due to accelerated elution and insufficient analyte-stationary phase equilibration, resulting in poorer peak definition.

In contrast, no significant secondary interactions were identified for AVO, indicating that its chromatographic behavior is influenced primarily by the individual factors rather than their combined effects. This distinction highlights that TBPT exhibits more complex interactions with the evaluated method parameters, especially for retention time (R1) and peak symmetry (R4), whereas AVO shows a more predictable and linear response to changes in experimental conditions.

Although the isolated and combined effects of the factors could be evaluated separately, an additional optimization step was conducted to ensure the overall method’s robustness. Using the selected chromatographic conditions, 84% organic phase concentration, 1 mL min^–1^ flow rate, and an oven temperature of 30 °C, the acceptable operational ranges for each factor were established based on their ability to maintain system suitability within the limits described in Table 1. These robustness ranges, which define the acceptable variability for routine use of the method, are summarized in Table 7.

**Table 7:** Robustness Ranges Established for the Critical Chromatographic Parameters Based on the Box–Behnken Design Optimization^a^

Factor	Robustness Range
Organic phase concentration (ACN:IPA %)	83.87–84.30
Oven temperature (°C)	25.42–31.91
Mobile phase flow rate (mL/min)	0.998–1.002

a ACN: acetonitrile; IPA: isopropanol.

The robustness evaluation allowed the definition of acceptable operating ranges for the critical chromatographic parameters without compromising system suitability or analytical performance. As shown in Table 7, the organic phase concentration exhibited a narrow robustness interval (83.87–84.30%), indicating high sensitivity of the method to changes in solvent strength, particularly for TBPT retention and peak symmetry. Similarly, the flow rate demonstrated a very tight tolerance window (0.998–1.002 mL min^–1^), reinforcing its strong influence on retention time, peak area, and overall separation efficiency. In contrast, oven temperature displayed a broader acceptable range (25.42–31.91 °C), suggesting that analyte viscosity and diffusivity are less susceptible to minor thermal fluctuations under the selected chromatographic conditions. Altogether, these ranges confirm that the method remains stable and reliable when operated within these limits, ensuring consistent simultaneous quantification of AVO and TBPT in complex matrices.

Moreover, the detailed regression equations and statistical parameters used to support the robustness optimization, including *R* ^2^, adjusted *R* ^2^, predicted *R* ^2^, *p*-values, *F*-values, adequate precision, lack of fit, and model type, are summarized in Table 8. These metrics provide a comprehensive assessment of the predictive ability and reliability of each fitted model.

**Table 8:** Regression Equations, Applied Models, and Statistical Parameters (*R* ^2^, Adjusted *R* ^2^, Predicted *R* ^2^, *p*-values, *F*-values, Adequate Precision, and Lack of-Fit) Obtained for the Robustness Evaluation of Avobenzone and Tris-Biphenyl Triazine^a^

Molecule	Response	Model	R^2^	Adjusted R^2^	Predicted R^2^	*F*-value	Adequate precision	Lack of fit	*p*-value
Avobenzone	R1	Quadratic	0.994	0.991	0.982	285.98	59.33	57.69	<0.0001
	R2	Linear	0.689	0.617	0.351	9.58	8.67	533.77	0.0013
	R3	Quadratic	0.964	0.948	0.893	58.73	22.02	19.23	<0.0001
	R4	Linear	0.596	0.569	0.495	22.12	9.70	9.57	0.0003
	**Regression equations**								
	$$R1=114.26-2.58\times A-0.09\times B+11.00\times C-0.18x\left(A\times C\right)+0.02\times A^{2}+0.00136\times B^{2}$$								
	R2=−46122.17+719.31×A+0.97×B−1683.47×C								
	$$R3=-52962.51+1297.81\times A+66.42\times B-1420\times C-7.68\times A^{2}-1.16\times B^{2}$$								
	R4=2.24−0.01×A								
Tris-biphenyl triazine	R1	Quadratic	0.997	0.996	0.990	618.51	83.44	11.35	<0.0001
	R2	Quadratic	0.933	0.911	0.837	41.98	23.10	12.17	<0.0001
	R3	Linear	0.935	0.930	0.915	214.74	30.21	17.52	<0.0001
	R4	2FI	0.539	0.432	–0.536	5.06	9.47	20.18	0.0154
	**Regression equations**								
	$$R1=3881.61-85.69\times A-2.68\times B-206.10\times C+0.03\times \left(A\times B\right)+2.21\times \left(A\times C\right)+0.48\times A^{2}$$								
	$$R2=1312683.16-30982.67\times A+141,14\times B-9454\times C+185.33\times A^{2}$$								
	R3=4320.98−2172.61×C								
	R4=−2.44+0.04×A+0.13×B−0.00155×(A×B)								

a R1: retention time in minutes; R2: number of theoretical plates; R3: peak area; R4: peak symmetry.

The statistical parameters presented in Table 8 confirm that the selected models were appropriate and statistically significant for most responses evaluated in the robustness study. For TBPT, retention time (R1), theoretical plates (R2), peak area (R3), and peak symmetry (R4) displayed strong or acceptable model fits, with *R* ^2^ values ranging from 0.539 to 0.997. In particular, the quadratic models applied to R1 and R2 demonstrated excellent predictive performance (*R* ^2^ > 0.93, predicted *R* ^2^ > 0.83), indicating that the selected factors and their interactions reliably explained the observed variability. The linear model for R3 also exhibited high predictive accuracy (*R* ^2^ = 0.935), confirming the flow rate as the predominant factor affecting the peak area. Although the 2FI model for R4 exhibited lower predictive ability (predicted *R* ^2^ < 0), the model remained statistically significant and aligned with the observed minimal sensitivity of peak symmetry to the studied factors.

For AVO, the model fits varied by response. Retention time (R1) and peak area (R3) were well described by quadratic models, with high *R* ^2^ and predicted *R* ^2^ values (*R* ^2^ ≥ 0.964), indicating a strong predictive capability. In contrast, linear models were sufficient for theoretical plates (R2) and peak symmetry (R4), albeit with lower predictive power (*R* ^2^ = 0.596–0.689), which is consistent with earlier observations that AVO exhibits minimal sensitivity to changes in chromatographic conditions. The lack of fit was statistically significant for most models; however, given the structured nature of response surface designs and the high adequate precision values (≥8.67 for all models), the models were still appropriate for defining robustness criteria.

Collectively, the regression analyses confirm that the applied models accurately characterize how chromatographic parameters affect the analyte behavior. These results further support the robustness ranges established in Table 7 and reinforce the method’s reliability under small, deliberate variations in analytical conditions.

Considering all of the obtained results, to the best of our knowledge, this study presents the first reported isocratic HPLC method for the simultaneous determination of AVO with other UV filters such as TBPT, addressing an important gap in the literature. Existing analytical approaches predominantly focus on the isolated quantification of each compound or on combinations of AVO with other UV filters, such as oxybenzone, octinoxate (OMC), or octocrylene, most of which require gradient elution for multicomponent separation. For TBPT, available methods typically pair it with polysilicone-15 or other triazine derivatives with no reported integration alongside AVO.

The simultaneous analysis developed in this study enables single-run quantification of both AVO and TBPT, markedly improving the analytical efficiency for routine quality control of sunscreen formulations, particularly when these filters are combined synergistically to enhance photostability. The method produced sharp and symmetrical chromatographic peaks with retention times of 4.24 min for AVO and 19.18 min for TBPT, resulting in a total runtime of approximately 20–25 min, which is competitive with and in many cases shorter than reported gradient-based methods for similar analytes.

Most HPLC methods described in the literature for AVO focus on its combination with UVB filters, such as an OMC, or with antioxidants, and do not extend to **triazine-based UV filters**. For example, Martins et al. (2022)^15^ reported an isocratic method for the simultaneous determination of AVO, OMC, and rutin; however, this approach does not address highly hydrophobic triazine filters and was primarily developed to investigate cyclodextrin-related matrix effects rather than routine sunscreen analysis.

To date, the study by Scarpin et al. (2021)^8^ represents the only reported HPLC-based method that includes TBPT within its analytical scope. In that work, a gradient elution method was employed for photostability and phototoxicity studies involving AVO and TBPT, using **long runtimes (>40 min)** and complex solvent systems, which limit its applicability for routine quality control environments.^8^

A comparative overview of the proposed method and representative HPLC methods reported for AVO and triazine-related UV filters is provided in Table 9.

**Table 9:** Comparison of the Proposed Method with Representative HPLC Methods for Avobenzone- and Triazine-Related UV Filters^a^

Method	UV filters analyzed	Elution mode	Runtime (min)	AVO and triazine-related retention time (min)	LOD/LOQ (μg mL^–1^)	Matrix	Main limitation
Martins et al., 2022 ^15^	AVO, OMC, rutin	Isocratic	∼22	AVO: 20.22	∼1–2	Cyclodextrin systems	No triazine-based filters
				TBPT: Not applicable			
Dencausse et al., 2008 ^14^	AVO, EMT, BZ-3, OMC	Gradient	∼30	AVO: ∼20.4	Not reported	Sunscreen emulsions	Gradient elution; THF-containing mobile phase; does not address TBPT
				EMT: ∼29.4			
Scarpin et al., 2021 ^8^	AVO, OMC, TBPT, RP, EHMCR	Gradient	>40	AVO: ∼19 (estimated)*	Not reported	Sunscreen formulations	Long runtime, complex gradient
				TBPT: ∼ 38 (estimated)*			
Proposed method	AVO, TBPT	Isocratic	∼20–25	AVO: 4.24	0.49–1.50	Lab-formulated sunscreen emulsion	Designed for robustness, not ultrafast
				TBPT: 19.18			

a AVO: avobenzone; OMC: octinoxate; TBPT: tris-biphenyl triazine; RP: retinyl palmitate; EHMCR: ethylhexyl methoxycrylene; BZ-3: benzophenone-3; EMT: bis-ethylhexyloxyphenol methoxyphenyl triazine; THF: tetrahydrofuran. * Retention times for avobenzone and tris-biphenyl triazine in Scarpin et al. (2021) were not numerically reported in the paper. They were therefore estimated from the chromatographic profile provided by the authors.

Retention time data further emphasize the analysis-oriented positioning of the proposed approach. In the isocratic method reported by Martins et al. (2022),^15^ AVO elutes at approximately 20.22 min, reflecting its strong hydrophobic interaction with the stationary phase. In the gradient method described by Scarpin et al. (2021),^8^ AVO and TBPT elute at approximately 19 and 38 min, respectively, as estimated from the reported chromatographic profiles. In contrast, the proposed isocratic method achieves significantly earlier elution for AVO (4.24 min) while maintaining the effective separation of the highly hydrophobic TBPT (19.18 min), demonstrating its ability to efficiently handle compounds with markedly different hydrophobicities.

In addition to Scarpin et al. (2021),^8^ Dencausse et al. (2008)^14^ developed a validated gradient HPLC method for the simultaneous determination of AVO and the triazine UV filter bis-ethylhexyloxyphenol methoxyphenyl triazine (EMT, Tinosorb S) in high-protection-factor emulsions. In that study, retention times of approximately 20.4 min for AVO and 29.4 min for EMT were achieved using a ternary gradient system comprising tetrahydrofuran (THF), acetonitrile, and aqueous acetic acid. Although this work demonstrates the feasibility of chromatographic separation of triazine derivatives, the **complexity of the gradient system**, the use of THF, and the lack of applicability to **highly hydrophobic triazines such as TBPT** represent important limitations.

It is important to emphasize that the aim of the present method was **not to compete with ultrafast or explicitly green chromatographic approaches**, but rather to establish a **robust, reproducible, and widely accessible isocratic method** suitable for routine quality control and formulation development. Triazine-based UV filters such as TBPT are inherently highly hydrophobic and, thus, strongly retained on reversed-phase stationary phases, regardless of the elution mode. Consequently, method robustness and reproducibility were prioritized over aggressive runtime minimization to ensure reliable quantification in complex sunscreen matrices.

Furthermore, several literature reports highlight substantial challenges associated with **matrix effects in sunscreen formulations**, often necessitating labor-intensive sample-preparation procedures such as pressurized liquid extraction or solid-phase extraction. In contrast, the method developed in this study offers a **simpler, reliable, and robust analytical alternative**, capable of directly handling formulations containing both AVO and TBPT simultaneously. This characteristic reinforces its suitability for **formulation development, stability studies, and routine quality control applications** involving complex sunscreen matrices.

## Conclusion

The simultaneous determination of highly hydrophobic UV filters in complex sunscreen matrices remains an analytical challenge, particularly for triazine-based compounds. In this study, an **isocratic HPLC method** for the simultaneous determination of avobenzone (AVO) and tris-biphenyl triazine (TBPT) was successfully developed and validated. The method was intentionally designed to be **robust, simple, and widely accessible**, and its performance was demonstrated in full accordance with **ICH Q2­(R1)**, exhibiting adequate specificity, linearity, sensitivity, accuracy, precision, and robustness.

Method robustness was further supported by a **Quality-by-Design (QbD)** approach using a **Box–Behnken Design**, enabling the systematic evaluation of critical chromatographic parameters and definition of a reliable analytical domain. Applicability was demonstrated using a **representative laboratory-prepared sunscreen formulation**, which effectively simulated real formulation complexity and confirmed the method’s suitability for routine quantitative analysis and quality control purposes.

Overall, the proposed method provides a **practical and reproducible analytical tool** for routine analysis and formulation development of sunscreen products. Importantly, it is well positioned to support **current and future research and quality control needs**, particularly in the context of evolving regulatory frameworks and the increasing approval of new-generation UV filters.

## Supplementary Material



## Notes

Representative chromatograms of avobenzone and tris-biphenyl triazine at 0.5, 1.0, and 2.0 μg mL^–1^, corresponding to concentrations near the detection and quantification limits, are provided for visual confirmation of method sensitivity (PDF)
