# Supplementary material for: Development and Validation of an Isocratic HPLC Method for the Simultaneous Determination of Avobenzone and Tris-Biphenyl Triazine
Source: ACS Omega. 2026 Mar 12;11(13):20534–46. doi: 10.1021/acsomega.5c12064 (PMC13063171; doi:10.1021/acsomega.5c12064)
Supplement: Supplementary file 1 [file ao5c12064_si_001.pdf]

SUPPORTING INFORMATION

Development and Validation of an isocratic HPLC Method for the Simultaneous Determination of Avobenzone and Tris-Biphenyl Triazine

Júlio A. Miranda[1,2], Yasmin F. da Cruz[1], Éverton N. Alencar[3], Wógenes N. Oliveira[1], Daniel Cristian F. Soares[4], Maureen D. Donovan[2], E. Sócrates T. Egito[1,2,]*

[1]Graduate Program in Health Sciences, Federal University of Rio Grande do Norte, UFRN, Natal, RN, Brazil;

[2]Department of Pharmaceutical Sciences and Experimental Therapeutics, College of Pharmacy, University of Iowa, Iowa City, IA, United States of America;

[3]Laboratory of Micro and Nanostructured Systems, College of Pharmaceutical Sciences, Food and Nutrition, Federal University of Mato Grosso do Sul (UFMS), Campo Grande, MS, Brazil;

[4] Laboratory of Bioengineering, Pure and Applied Institute, Federal University of Itajuba, Itabira, MG, Brazil.

*Corresponding author: socratesegito@gmail.com; Tel.: +55 (84) 9 9431 8816

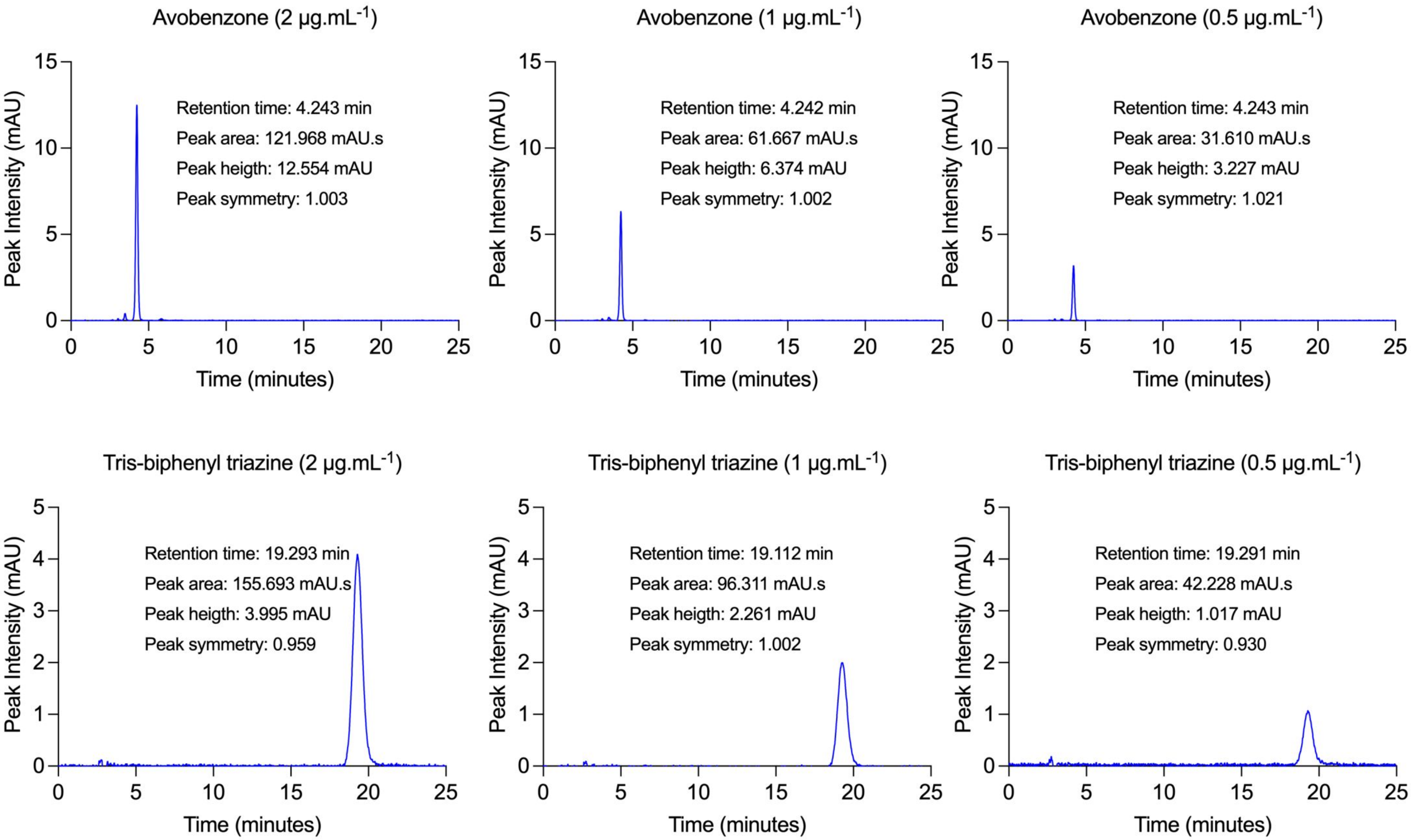

Figure S1. Representative chromatograms of avobenzone and tris-biphenyl triazine at 0.5, 1.0, and 2.0 μg mL^{-1}, corresponding to levels near the limit of detection (0.5 μg mL^{-1}) and the limit of quantification (1.0, and 2.0 μg mL^{-1}).
